# Identifying alemtuzumab as an anti-myeloid cell antiangiogenic therapy for the treatment of ovarian cancer

**DOI:** 10.1186/1479-5876-7-49

**Published:** 2009-06-19

**Authors:** Heather L Pulaski, Gregory Spahlinger, Ines A Silva, Karen McLean, Angela S Kueck, R Kevin Reynolds, George Coukos, Jose R Conejo-Garcia, Ronald J Buckanovich

**Affiliations:** 1 Department of Obstetrics and Gynecology, University of Michigan, Ann Arbor, USA; 2 Department of Internal Medicine, University of Michigan, Ann Arbor, USA; 3 Department of Obstetrics and Gynecology, University of Pennsylvania, Philadelphia, USA; 4 Departments of Microbiology and Immunology, Dartmouth Medical School, Hanover, USA

## Abstract

**Background:**

Murine studies suggest that myeloid cells such as vascular leukocytes (VLC) and Tie2^+ ^monocytes play a critical role in tumor angiogenesis and vasculogenesis. Myeloid cells are a primary cause of resistance to anti-VEGF therapy. The elimination of these cells from the tumor microenvironment significantly restricts tumor growth in both spontaneous and xenograft murine tumor models. Thus animal studies indicate that myeloid cells are potential therapeutic targets for solid tumor therapy. Abundant VLC and Tie2^+ ^monocytes have been reported in human cancer. Unfortunately, the importance of VLC in human cancer growth remains untested as there are no confirmed therapeutics to target human VLC.

**Methods:**

We used FACS to analyze VLC in ovarian and non-ovarian tumors, and characterize the relationship of VLC and Tie2-monocytes. We performed qRT-PCR and FACS on human VLC to assess the expression of the CD52 antigen, the target of the immunotherapeutic Alemtuzumab. We assessed Alemtuzumab's ability to induce complement-mediated VLC killing in vitro and in human tumor ascites. Finally we assessed the impact of anti-CD52 immuno-toxin therapy on murine ovarian tumor growth.

**Results:**

Human VLC are present in ovarian and non-ovarian tumors. The majority of VLC appear to be Tie2+ monocytes. VLC and Tie2+ monocytes express high levels of CD52, the target of the immunotherapeutic Alemtuzumab. Alemtuzumab potently induces complement-mediated lysis of VLC in vitro and ex-vivo in ovarian tumor ascites. Anti-CD52 immunotherapy targeting VLC restricts tumor angiogenesis and growth in murine ovarian cancer.

**Conclusion:**

These studies confirm VLC/myeloid cells as therapeutic targets in ovarian cancer. Our data provide critical pre-clinical evidence supporting the use of Alemtuzumab in clinical trials to test its efficacy as an anti-myeloid cell antiangiogenic therapeutic in ovarian cancer. The identification of an FDA approved anti-VLC agent with a history of clinical use will allow immediate proof-of-principle clinical trials in patients with ovarian cancer.

## Introduction

There is increasing evidence that monocyte derived myeloid cells expressing vascular markers such as Tie2 or VE-Cadherin support tumor growth [1-5]. These cells are recruited to regions of hypoxia and promote angiogenesis and vasculogenesis [6,7]. Myeloid cell recruitment to the tumor bed appears to precede or coincide with the 'angiogenic switch'[8,9]. In an established tumor, myeloid cells appear to be a primary source of resistance to anti-VEGF therapy, suggesting a critical role for these cells in tumor angiogenesis [5].

The exact mechanism of action of myeloid cells remains contentious. These cells can clearly promote angiogenesis through local production of angiogenic factors[1,10-13]. Some studies have suggested that these cells may be able to trans-differentiate to assume an endothelial cell fate, incorporate into vessel lumens, and contribute to vasculogenesis[3,14-17].

While the exact function of these proangiogenic myeloid cells remains controversial, murine studies confirm a critical role for these cells in tumorigenesis and indicate that these cells may be novel therapeutic targets for solid tumor therapy. Genetic manipulations to inhibit or eliminate these cells in both spontaneous and xenograft murine tumor models can severely restrict tumor growth [3,7,9,18]. Similarly, therapeutics targeting these cells reduce microvascular density and restrict tumor growth [15,19].

Proangiogenic myeloid cells similar to those found in mice have also been identified in human tumors. Myelo-monocytic cells expressing the hematopoietic marker CD14 and various vascular markers such as Tie2 (Tie2^+ ^Monocytes), VE-Cadherin, and VEGFR2 have been reported to take part in both ischemia-associated and tumor-associated angiogenesis [17,20]. We reported the presence of a proangiogenic myeloid cell population, expressing numerous myeloid (CD14, CD45, CD11c, CD11b) and vascular (VE-Cadherin, CD31, CD146) surface markers, in ovarian cancer [21]. Given the dual phenotype of these cells, expressing both myeloid and vascular specific markers, and an angiogenic phenotype, we have termed these cells vascular leukocytes (VLC) [15,21]. VLC represent 10–70% of host cells and up to 30% of all cells in ovarian cancer ([21] and unpublished data. In vitro and in vivo studies indicate VLC play a role in tumor angiogenesis. Increased recruitment of VLC to tumors by the chemokine B-Defensin-29 significantly increased murine tumor growth [15]. Similarly, the direct addition of VLC to human tumor xenografts increased tumor microvascular density. VLC produce numerous pro-angiogenic factors such as TGF-β, VEGF, and Interleukin-8. VLC promote endothelial tubulogenesis and participate in perfusable vascular structures in matrigel in vivo [15,21,22]. Importantly, inhibiting or eliminating VLC or similar myeloid cells in mice inhibits angiogenesis and severely restricts tumor growth [15,19].

Similar to VLC, proangiogenic CD14^+^/Tie2^+ ^monocytes have recently been reported to be present in human tumors [20]. Tie2^+ ^monocytes were identified in low numbers in the peripheral blood of cancer patients. Like VLC, Tie2^+ ^monocytes are present in high numbers in tumor tissue, but are rare in normal tissue. Also similar to VLC, the addition of Tie2^+ ^monocytes (but not Tie2-depleted monocytes) to tumor xenografts enhanced tumor microvascular density [23]. Tie2^+ ^monocytes were described in many solid tumors including colon, lung, renal and breast cancer.

As animal studies indicate that VLC and Tie2^+ ^monocytes are potentially legitimate therapeutic targets for solid tumor therapy, we sought to determine the relationship of VLC and Tie2^+ ^monocytes. Furthermore, we attempted to identify an anti-VLC therapeutic for use in human cancers. We demonstrate here that many VLC appear to be a subset of Tie2^+ ^monocytes. We identify the expression the hematopoietic antigen CD52, the target of the immunotherapeutic Alemtuzumab, on human VLC and Tie2^+ ^monocytes. We show that Alemtuzumab is capable of inducing complement-mediated VLC killing. Finally, anti-VLC therapy with an anti-CD52 immunotoxin significantly restricted ovarian tumor growth in a murine ovarian tumor model. These studies provide important pre-clinical data supporting the use of Alemtuzumab as a therapeutic agent for ovarian cancer patients.

## Materials and methods

### Tissues

Stage III epithelial ovarian cancer (n = 10), and ductal breast cancer specimens (n = 1), non-small cell lung carcinoma (n = 3) (provided by Dr. Steven M. Albelda and Dr. Doug Arenberg) and melanoma (n = 3) (provided by Dr. David Elder), normal ovary (n = 2) and normal endometrium (n = 2) were collected at the University of Pennsylvania or the University of Michigan. After obtaining informed patient consent, ascites was collected either intraoperatively or at the time of therapeutic paracentesis. All specimens were processed in compliance with IRB and HIPAA requirements.

### Tumor Processing

Freshly harvested solid tumors were mechanically dissected into 1–2 mm pieces and then further isolated to single cells using the Medi-machine (BD Pharmingen). Cell suspensions were then passed through a 40 um filter and finally isolated on ficoll gradient as previously described [21].

### Ascites Processing

For FACS characterization of VLC, ascites associated cells were concentrated by centrifugation and then red blood cells were lysed using ACK buffer (lonza, Walkersville, MD. Host cells were then isolated using a Ficoll gradient. Cells were then passed through a 40 um filter followed by 4 passes through a 28G needle to isolate single cells for FACS. For Alemtuzumab induced cytotoxicity assays in whole ascites, after red cell lysis, whole cell pellets were resuspended in 1/20^th ^of the original volume of ascites supernatant and used directly in cytotoxicity assays.

### FACS

Human CD45^+^/VE-Cadherin^+ ^(CD144) vascular leukocytes and CD45^(-)^/VE-Cadherin^+ ^tumor endothelial cells were FACS isolated from the ficoll isolated cells using APC anti-CD45 (BD Pharmingen, San Diego, CA) and PE-mouse anti-human CD144 antibody (eBioscience, San Diego, CA). CD52 expression was confirmed with using FITC-anti-human CD52 (GeneTex San Antonio, TX). For qRT-PCR experiments, a second vascular marker CD146 (P1H12-eBiosciences), was used in conjunction with CD45 and VE-Cadherin to increase purity.

Tie2 expression was confirmed using biotin-anti-human-Tie2 (Abcam Cambridge, MA) coupled with streptavidin-FITC. Tie2 monocytes were characterized using mouse anti-CD14-FITC (BD Pharmingen) and Mouse anti-human Tie2-APC (R&D Systems Minneapolis, MN). VE-Cadherin expression on Tie2 monocytes was confirmed using anti-VE-Cadherin-PE antibody. In order to avoid nonspecific antibody binding, PBS containing 10% normal murine serum (Sigma, St. Louis, MO) and 25 μg/ml anti-mouse Fc receptor (2.4G2 BD Pharmingen) were added prior to incubation. Mouse VLC were characterized using anti-CD45-APC (BD Pharmingen), anti-CD14-FITC and anti-CD14-PE (BD Pharmingen), anti-VE-Cadherin-biotin (Bender-Medsystems), and anti-CD52-PE (MBL, Cambridge, MA).

### Complement-mediated Cytotoxicity of Isolated VLC

VLC FACS-isolated from ovarian tumor as described above were incubated with 10 μg/ml of Alemtuzumab (Genzyme Cambridge, MA) for thirty minutes. Isolated VLC were washed and incubated with 10% human serum or heat inactivated serum at 37°C for one hour (human serum was inactivated by incubating at 60°C for thirty minutes immediately prior to use). CD3+ peripheral blood lymphocytes were used as a positive control. Cells were then stained with Annexin-FITC (BD Pharmingen) and propidium iodide (BD Pharmingen) per manufacturer's protocol. To assure cellular viability throughout the assay, an aliquot of untreated VLCs were maintained in culture for the duration of the experiment. These untreated VLCs were stained for Annexin-V/PI in parallel with Alemtuzumab treated cells +/- inactivated serum. Cells negative for both Annexin V and PI were deemed viable cells.

### Complement-mediated Cytotoxicity of Whole Ascites

A single cell suspension of whole ascites cells (host and tumor cells) suspended in ascites fluid was incubated for 90 minutes with 10 μg/ml Alemtuzumab or heat inactivated Alemtuzumab (heated at 80°C for 30 minutes). Cells were then immediately labeled with anti-CD45-APC (BD Pharmingen) and anti-VE-Cadherin-PE (eBioscience), or Annexin-FITC and 7-Amino Actinomycin D (7-AAD BD Pharmingen) and analyzed by FACS. Once again to assess cellular viability an aliquot of cells which receive no treatment were maintained at 37C in the ascites fluid throughout the course of the experiment. Viability of this control aliquot was then assessed with AnnexinV and 7AAD. AnnexinV(-)/PI(-) cells were considered viable

### Quantitative RT-PCR

RNA was isolated from fresh VLC using the TRIzol method. RNA was reverse-transcribed into cDNA using superscript III per manufacturer's directions (Invitrogen Carlsbad, CA) and quantitative PCR was performed using 2 ng of total cDNA and SYBRgreen (Applied Biosystem; CD52, 5'primer CTTCCTCCTACTCACCATCAGC, 3'primer CCACGAAGAAAAGGAAAATGC).

### Histology

Immunofluorescence was performed on fresh frozen, acetone fixed tissue using an anti-CD52 antibody (1:100 GeneTex, Inc) and anti-VE-Cadherin FITC antibody (1:200 Bender MedSystems). Immunohistochemistry was performed on murine tumors with anti-CD31 antibody (1:800 BD Pharmingen) and vecta-stain (Vector Labs Burlingame, CA) per protocol as described by the manufacturer.

### CD52 Immunotoxin Development

Anti-CD52 antibodies (MBL Cambridge, MA) were biotinylated per protocol (Pierce). Biotinylation was confirmed by FACS analysis of murine splenocytes using biotinylated anti-CD52 antibody coupled with streptavidin-PE conjugate (BD Pharmingen). After biotinylation was confirmed, streptavidin-saporin (Advances Targeting Systems, San Diego, CA) was incubated with biotin labeled anti-CD52 antibodies in a 1.5:1 molar concentration. 2 μg/ml anti-CD52-saporin conjugate was then incubated with isolated ascites-associated cells for 36 hours in vitro and cytotoxicity confirmed by trypan blue and FACS staining (data not shown). To confirm in vivo toxicity, tumor bearing animals were treated twice-weekly with 2 ug of anti-CD52-saporin antibodies (n = 5) or control antibody (n = 3). After three weeks peripheral blood was collected, RBCs were lysed with ACK buffer, and then PBMCs were analyzed by FACS. Similarly tumors were resected, processed into single cells as described above and analyzed for VLC by FACS. Finally tumor ascites-bearing animals were treated with 2 μg of CD52-saporin or control IgG-saporin (n = 5 per group) daily for 48 hours and then ascites cells were harvested, red cells were lysed using ACK buffer, and whole ascites cell samples were analyzed for VLC by FACS.

### Treatment of Flank Tumors

20 × 10^6 ^ID8-VEGF cells were injected subcutaneously into the flanks of C57BL6 mice and the tumors were allowed to grow for two weeks. The animals were then treated twice weekly with 2 μg of anti-CD52-saporin immunotoxin, or rat-IgG-saporin or immunopurified rabbit IgG-saporin control (a total n = 10, n = 5 and n = 5 respectively, in two independent experiments). Immunotoxins were administered intraperitoneally twice-weekly for three weeks. Rat and rabbit immunoglobulin controls revealed similar results and are presented as pooled data. Tumor growth curves were analyzed using ANOVA and Student's t-test At the time of sacrifice a subset of animals were perfused with biotinylated lycopersicon esculentum (tomato) lectin as previously described [21].

### Treatment of Intraperitoneal Tumors

10 × 10^6 ^ID8 cells were injected intraperitoneally into C57BL6 mice randomized by weight. Starting one week after the injection of tumor cells, mice were treated with 2 μg of anti-CD52-saporin immunotoxin or rat-IgG-saporin (n = 10 per group in two independent experiments) twice-weekly for three weeks. Animals were weighed to assess tumor growth. Animals were euthanized when they demonstrated 10 gm of weight gain secondary to ascites or animals appeared moribund. Survival curves were compared with the log-rank statistic.

### Microvascular Density Analysis

CD31 IHC was performed simultaneously on four representative sections from 4 flank tumors in the treatment and control groups. Each section was systematically photographed in neighboring 40× fields such that 80–100% of each tumor section was photographed. Total CD31 stain area, as defined by pixel density and hue, was assessed using Olympus Microsuite Biological Suite software. Area of staining was then compared between control and treatment groups using a two-sided student's t-test.

## Results

### VLC are found in a variety of human solid tumors

**Figure 1:**
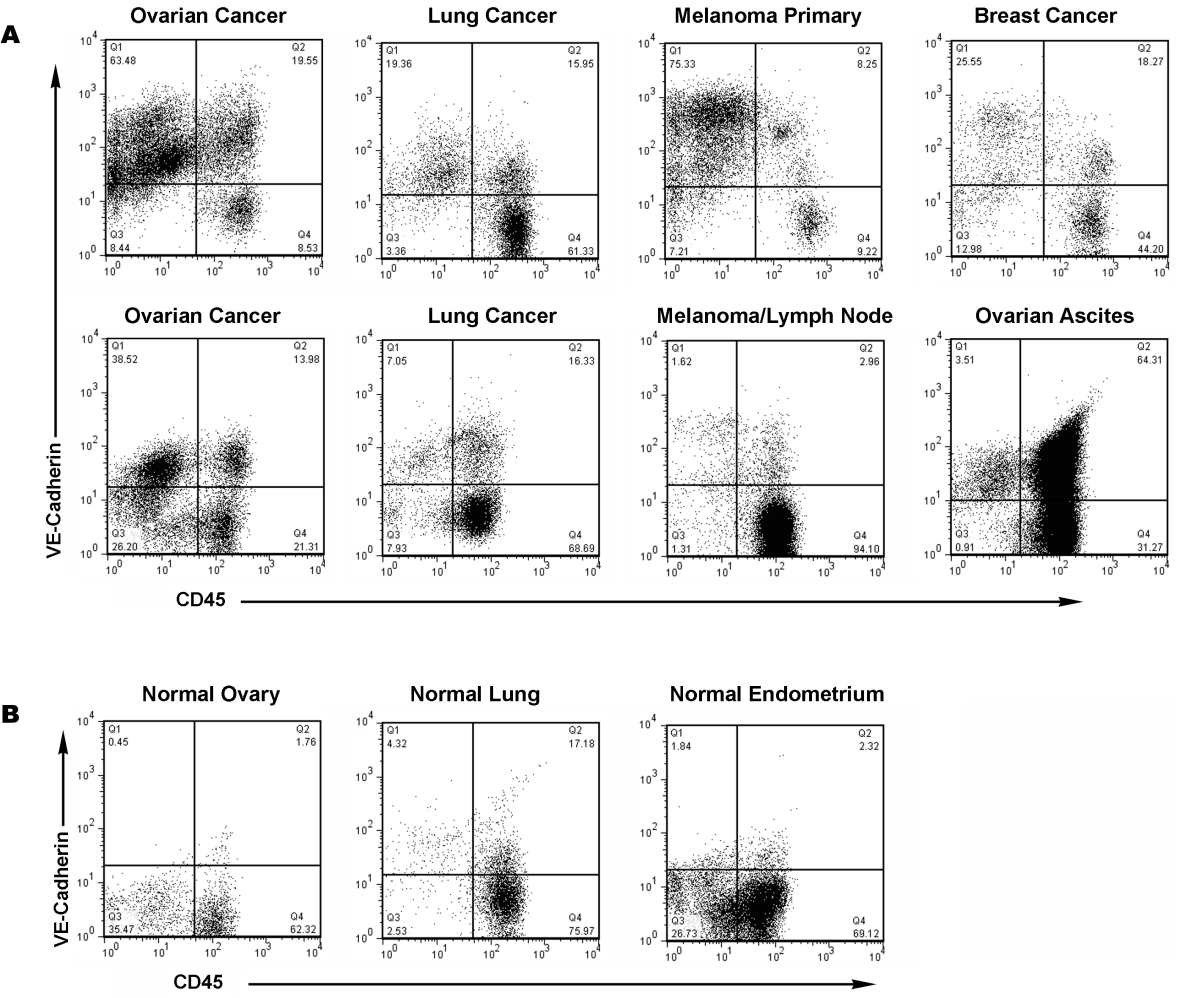
**VLC in tumor and normal tissues**. FACS analysis of VLC in (**A**) Ficoll isolated tumor associated host cells and (**B**) normal tissues as indicated. CD45 stain is indicated on the X-axis and VE-Cadherin stain is indicated on the Y-axis.

We have previously demonstrated significant numbers of CD45^+^/VE-Cadherin^+ ^VLC in stage III ovarian cancer solid tumors [21]. We tested whether these cells are unique to ovarian cancer or whether they are present broadly in human solid tumors. We used a ficoll gradient to isolate tumor associated host cells from mechanically dissociated surgical specimens of melanoma (n = 4), as well as breast (n = 1), lung (n = 8), and endometrial (n = 2) cancers. The presence of CD45^+^/VE-Cadherin^+ ^VLC in each tumor was assessed by flow cytometry (Figure 1A). VLC were present in all of the tumor samples analyzed, although in somewhat reduced numbers compared to ovarian cancer. Interestingly, very few VLC were observed in lymph nodes with metastatic melanoma (Figure 1A), suggesting VLC may not play a significant role in tumor growth within lymph nodes.

Similar to ovarian cancer, VLC isolated from melanoma, breast, lung, or endometrial cancer expressed endothelial markers such as CD146 and CD31, and myeloid markers such as CD14 (data not shown). Interestingly, a higher frequency of VLC was also found in normal lung tissue adjacent to lung adenocarcinoma, indicating that VLC may also accumulate in peritumoral host tissue. Lastly, VLC were found at low frequency in normal reproductive organs including ovary and endometrium (Figure 1B). Thus, VLC are found in many solid tumors and are not unique to ovarian cancer. Furthermore, they are found in normal tissue surrounding cancer and in some normal tissues that exhibit physiologic angiogenesis.

### VLC express CD52, the target of the immunotherapeutic Alemtuzumab

**Figure 2:**
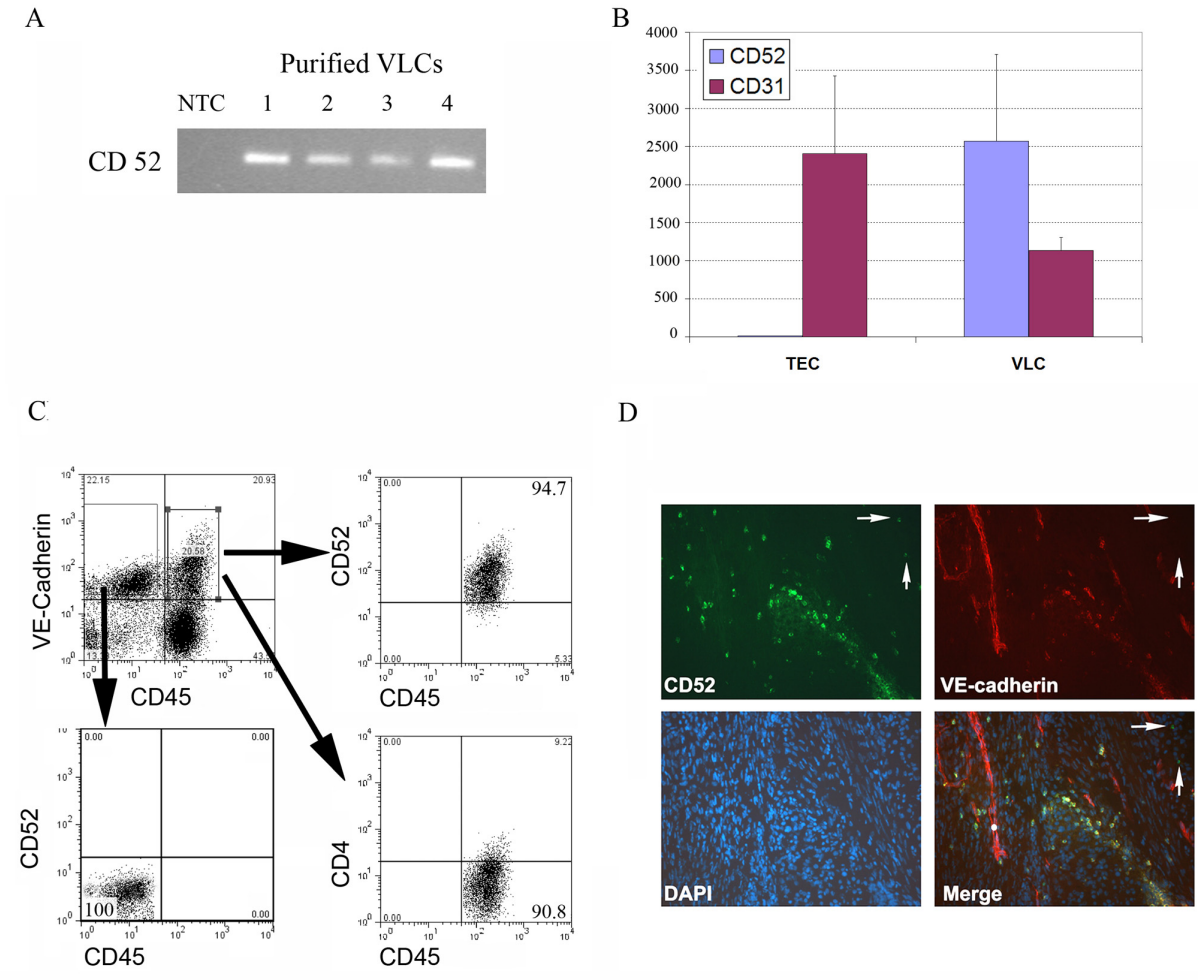
**VLC express CD52**. **A**. RT-PCR demonstrating CD52 mRNA expression in VLCs FACS isolated from 4 ovarian tumors (NTC-no template control). **B**. qRT-PCR quantification of CD52 mRNA expression in FACS-isolated VLC and tumor endothelial cells (TECs). **C**. FACS analysis confirming CD52 protein expression on CD45^+^/VE-Cadherin^+ ^VLC. VLC do not express the T cell marker CD4. CD45^(-)^/VE-Cadherin^+ ^tumor endothelial cells do not express CD52. **D**. Immunofluorescence demonstrating co-expression of VE-Cadherin (red) and anti-CD52 (green) in ovarian cancer. Arrows indicate CD52^+^/VE-Cadherin^(-) ^lymphocytes.

As murine studies have indicated that VLC are potential therapeutic targets, we assayed VLC for the expression of antigens that have well-developed immunotherapeutics. We isolated RNA from CD45^+^/VE-Cadherin^+^/CD146^+ ^VLC isolated by FACS from 4 independent ovarian cancer specimens. CD146, a tumor endothelial cell marker expressed on VLC [21], was included to enhance the purity of the VLC isolation. RT-PCR and qRT-PCR revealed CD52 mRNA expression in all four VLC specimens (Figure 2A and 2B). While CD31 mRNA was readily detected, no CD52 mRNA expression was detected in CD45^(-)^/VE-Cadherin^+^/CD146^+ ^tumor endothelial cells (TECs). FACS analysis of ficoll isolated tumor infiltrating host cells confirmed CD52 protein expression on greater than 90% of CD45^+^/VE-Cadherin^+^VLC (range 88–98%, Figure 2C). The level of expression was similar to that seen on tumor infiltrating lymphocytes (data not shown). As a negative control, no expression of CD4 (a T cell markers) was seen on VLC (Figure 2C). CD52 protein was not expressed on CD45^(-)^/VE-Cadherin^+ ^TECs or tumor cells (Figure 2C and see below).

Co-immunofluorescence on fresh frozen human epithelial ovarian tumors identified large CD52^+^/VE-Cadherin^+ ^cells primarily in a perivascular location and in ovarian tumor stroma. This is similar to the localization reported for Tie^+ ^monocytes in other tumors[24] Small CD52^+^/VE-Cadherin^(-) ^cells, consistent with tumor infiltrating lymphocytes, were also observed (Figure 2D). CD52 was not detected in the tumor endothelium or tumor cells, consistent with the RT-PCR and flow cytometry data.

These results confirm the expression of the CD52 antigen on VLC. CD52 has been well established as an immunotherapeutic target antigen. In fact, an anti-human CD52 antibody therapy, Alemtuzumab (Campath) has been developed and is FDA approved for the treatment of CD52 expressing leukemia. Taken together, this data suggest Alemtuzumab may be used to target VLC in tumors.

### Alemtuzumab induces complement-mediated lysis of VLC in vitro and ex vivo in tumor ascites

**Figure 3:**
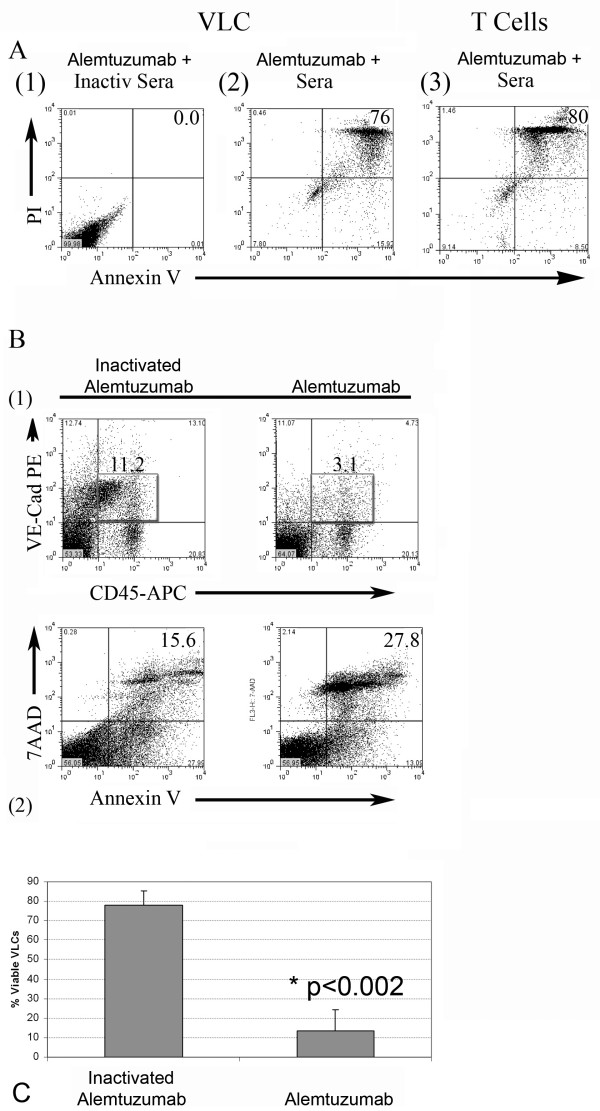
**Alemtuzumab induced complement-mediated cytotoxicity of VLC**. A VLCs FACS isolated from ovarian tumor tissue incubated with Alemtuzumab in the presence or absence of complement; **(1) **In the presence of Alemtuzumab and heat inactivated sera, the majority of VLC are viable Annexin V ^(-) ^and PI ^(-) ^cells. In contrast, in the presence of Alemtuzumab and sera **(2)**, the majority of VLC are Annexin V^+ ^and/or PI ^+ ^indicating the induction of cytotoxicity (n = 3). **(3) **In the presence of Alemtuzumab and sera, cytotoxicity was similarly induced in control CD3+ peripheral blood T cells. **B**. To determine if Alemtuzumab could induce cytotoxicity of VLC in whole tumor ascites ex vivo, we incubated ascites associated cells in ascites fluid together with either heat inactivated Alemtuzumab or Alemtuzumab. **(1) **In the presence of heat inactivated Alemtuzumab a population of CD45^+^/VE-Cadherin+ cells was clearly detectable (box). In contrast in the presence of active Alemtuzumab there is as significant reduction of VLC. **(2) **Loss of CD45^+^/VE-Cadherin^+ ^VLC in the presence of Alemtuzumab was associated with an appropriate increase in Annexin V/PI-labeled cells. **C**. Summary of Alemtuzumab anti-VLC activity from independent patient samples (n = 3) p = 0.002.

Alemtuzumab has been shown to induce death of CD52-expressing cells by complement-mediated cytotoxicity [25-27]. We sought to determine if Alemtuzumab could induce complement-mediated cellular cytotoxicity of isolated ovarian cancer VLC in vitro. In the absence of complement and Alemtuzumab, approximately 90% of purified VLC are viable as evidenced by the Annexin V ^(-)^/PI^(-) ^cells (Figure 3A(1) and data not shown). The addition of Alemtuzumab and human serum (as a complement source) to isolated VLC in vitro lead to a statistically significant induction of apoptosis and cell death, as defined by Annexin V and propidium iodide staining, in nearly 100% (range 76–99%, p < 0.001)) of VLC (Figure 3A(2) and data not shown). Identical results were obtained with CD3+ peripheral blood T cells (Figure 3A(3)). Consistent with complement-mediated cytotoxicity, heat inactivation of the sera lead to a considerable loss of Alemtuzumab's cytotoxic activity.

As the tumor microenvironment can be immunosuppressive and express complement inhibitors, we next sought to ascertain the ability of Alemtuzumab to kill VLC within a human tumor milieu. We added Alemtuzumab to freshly isolated tumor ascites/ascites-associated cells ex vivo. Whereas VLC were readily detectable in the presence of heat inactivated Alemtuzumab, 75% of VLC were eliminated in the presence of fresh Alemtuzumab (Figure 3B(1) and 3C). This was associated with a proportionate increase in the presence AnnexinV^+^/7-AAD^+ ^apoptotic cells (Figure 3B(2)). This indicates that Alemtuzumab can induce complemented-mediated cytotoxicity of VLC even within the tumor milieu and confirms the potential use of Alemtuzumab as an anti-VLC therapeutic in humans with ovarian cancer.

### A Majority of VLC are Tie2^+ ^monocytes

**Figure 4:**
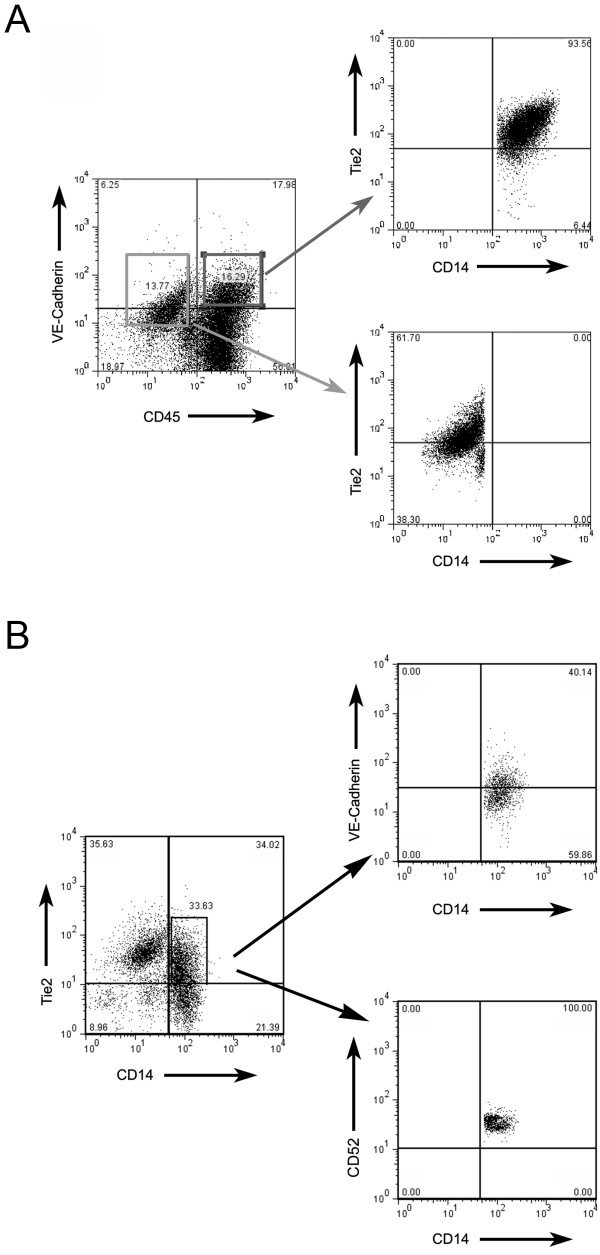
**VLC are Tie2+ monocytes**. FACS Analysis demonstrating, **A**. CD45^+^/VE-Cadherin^+ ^VLC (red box) are CD14^+^/Tie2^+ ^(Top right) and CD45^(-)^/VE-Cadherin^+ ^endothelial cells (blue box) are CD14^(-)^/Tie2^+ ^(bottom right). **B**. A portion of CD14^+^Tie2^+ ^cells are VE-Cadherin^+^. All CD14^+^/Tie2^+^cells are CD52^+^.

We previously reported that VLC were CD14^+ ^cells which express numerous endothelial markers [21]. More recent studies have reported a population of CD14^+ ^cells expressing the vascular marker Tie2 (Tie2^+ ^Monocytes) [6,20,23,24]. VLC and Tie2^+ ^monocytes appear functionally similar. We therefore performed FACS analysis of VLC to determine if VLC express Tie2. As expected, CD45^(-)^/VE-Cadherin^+ ^tumor endothelial cells were Tie2^+^/CD14^(-)^. In contrast, FACS demonstrated that the majority of CD45^+^/VE-Cadherin^+ ^VLC (64–90%) are Tie2^+ ^and CD14^+ ^(Figure 4). Thus by definition, the majority of VLC are Tie2^+ ^monocytes. Interestingly, only ~50% of CD14^+^/Tie2^+ ^cells (range 40–74%) were VE-Cadherin^+^. Thus, while the majority of VLC are Tie2 Monocytes, the majority of Tie2 monocytes are not necessarily VLC. As Alemtuzumab effectively eliminated nearly 100% of tumor associated VLC (Figure 3), Alemtuzumab is therefore capable of targeting at least some Tie2^+ ^monocytes. In addition,, FACS demonstrated that nearly all Tie2^+ ^monocytes (range 90–100%) are CD52^+^, indicating Alemtuzumab may target Tie2^+ ^monocytes independent of their relationship to VLC.

### Development of an anti-murine CD52 immunotoxin

**Figure 5:**
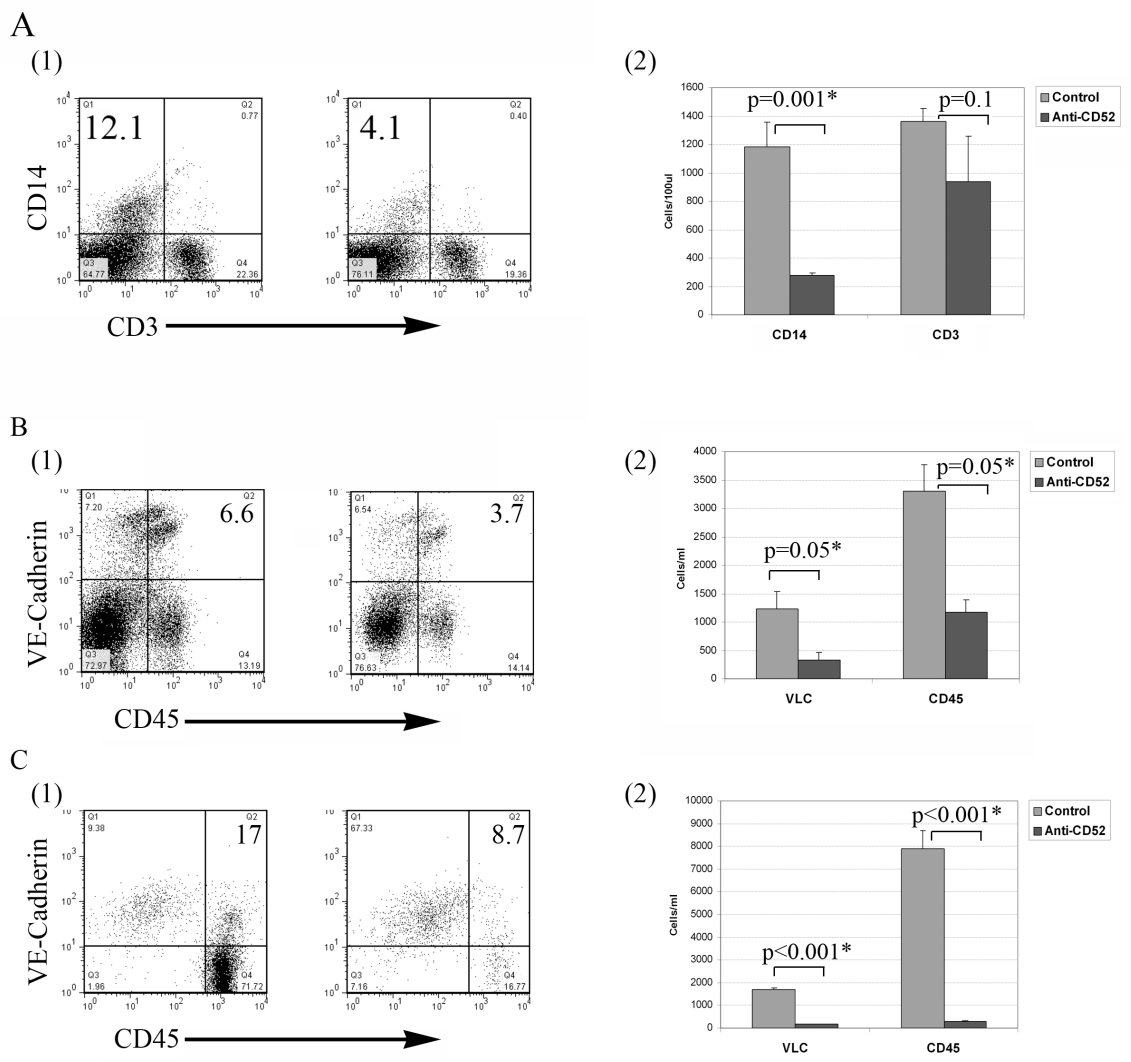
**Confirmation of activity of the murine anti-CD52 immunotoxin**. **A (1) **FACS analysis of CD14^+ ^and CD3^+ ^cells in peripheral blood mononuclear cells isolated from control (n = 3) and anti-CD52 immunotoxin treated mice (n = 5) demonstrating a reduction in the percentage of both CD14^+ ^and CD3^+ ^cells in treated animals. **A(2) **Quantification of absolute numbers of CD14^+ ^and CD3^+ ^cells in peripheral blood of control and anti-CD52 treated animals. **B (1 and 2) **Quantification of VLC percent and absolute number in tumor associated ascites of control and anti-CD52 immunotoxin treated animals (n = 5 per group). **C(1 and 2) **Quantification of VLC percent and absolute number in solid tumors of control (n = 3) and anti-CD52 immunotoxin treated animals (n = 5). Tumors were harvested immediately after discontinuation of therapy.

In order to test the effects of anti-CD52 antibody therapy on tumor growth in vivo, we developed an anti-CD52 immunotoxin. Unlike Alemtuzumab, murine anti-CD52 antibodies do not induce complement-mediated or antibody-dependent cellular cytotoxicity. We therefore coupled anti-murine CD52 antibodies with saporin toxin. Saporin immunotoxins have been well described and successful at targeting VLC[15,19] Anti-murine CD52-saporin was administered to tumor bearing mice twice-weekly for three weeks and then animals were sacrificed 24 hours after the last administration of the immunotoxin. Analysis of peripheral blood mononuclear cells demonstrated that anti-CD52 immunotoxin treated animals had a significant reduction in both CD14 and CD3+ cells (Figure 5A). Interestingly, the impact on CD14+ cells was greater than that seen on CD3+ cells. Similarly analysis of tumors revealed a significant reduction in VLC and CD45+ cells in both flank tumors and orthotopic tumors (ascites) models (Figure 5B and 5C).

### Anti-CD52 therapy restricts tumor growth in a murine model of ovarian cancer

**Figure 6:**
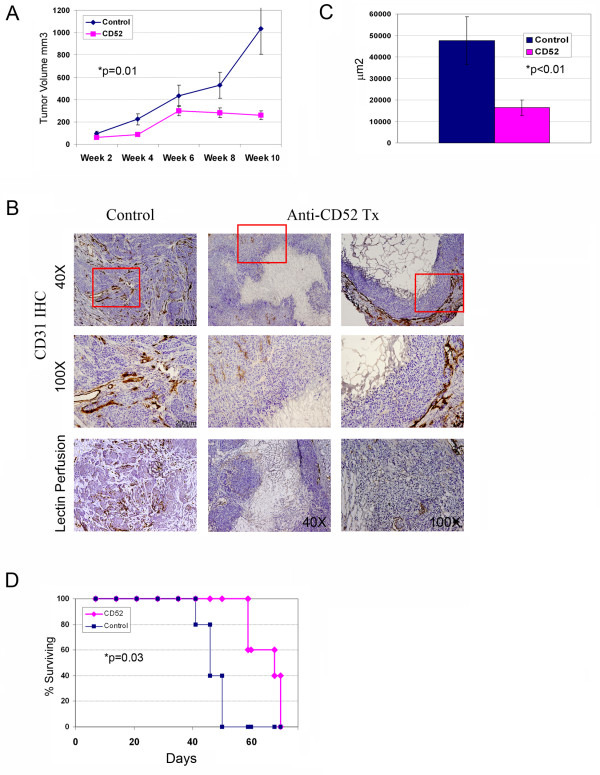
**Anti-CD52 therapy restricts tumor growth**. **A(1)**. Tumor growth curves for control and anti-CD52 treated (n = 10 per group in duplicate experiments) subcutaneous ID8-VEGF ovarian tumors. Tumor growth was significantly restricted with anti-CD52 therapy (p = 0.01). **B**. Representative sections of CD31 IHC and lectin perfusion labeling of ID8 flank tumors demonstrating significant reduction in tumor penetrating vessels and vascular perfusion in control and CD52-treated tumors. Magnification and scale bars as indicated. **C**. Quantification of microvascular density in anti-CD52 treated tumors and control tumors assessed by CD31 IHC. **D**. Kaplan Meier survival plots for control and anti-CD52 immunotoxin treated animals (n = 10/group) using an orthotopic intraperitoneal ID8 tumor model. Overall survival was significantly increased by anti-CD52 therapy (p = 0.03).

We next tested the impact of anti-CD52 antibody therapy on ovarian tumor growth in vivo using the ID8-VEGF murine ovarian flank tumor model. As above, animals with established tumors were treated with anti-CD52 therapy twice-weekly for three weeks. Therapy was then discontinued and tumor growth was monitored for several weeks. Therapy significantly restricted solid tumor growth throughout the course of the experiment (p < 0.05) (Figure 6A). Treatment of flank tumors was associated with a significant reduction in tumor microvascular density (Figure 6B and 6C). This reduction in microvascular density was also correlated with a reduction in tumor perfusion density (Fig 6B and 6C).

Finally, we used an orthotopic intraperitoneal model of ovarian cancer to assess the impact of therapy on animal survival. In this model animal reproducibly develop tumor associated ascites requiring euthanasia of the animals. ID8 cells were grown intraperitoneally inC57BL6 mice. Twice-weekly intraperitoneal anti-CD52 therapy was initiated one week after the injection of tumor cells. Anti-CD52 immunotoxin therapy lead to a delay in the accumulation of tumor-associated ascites and an improvement in the median overall survival of treated animals (Figure 6D). These results confirm the anti-tumor activity of anti-VLC therapy, as observed by others, and further support the use of anti-CD52 therapy in humans.

## Discussion

Our study adds to a growing body of literature indicating myeloid cells are legitimate therapeutic targets in the treatment of solid tumors. Several studies have used transgenic mice to demonstrate the importance of various myeloid cell populations. MMP-9 knockout mice were used to demonstrate a role for Gr^+^/CD11b^+ ^cells in tumor vascularization [3]. In fact, MMP-9 producing bone marrow derived cells have been implicated in both tumor angiogenesis and vasculogenesis [7,18]. Similarly, a transgenic suicide gene approach was used to demonstrate a potent anti-tumor effect of eliminating Tie+ monocytes [23]. While representing important proofs of concept, these techniques obviously cannot be applied to humans. Other studies have utilized immunotherapeutic approaches; antibody therapeutics targeting chemokine receptor-6 and scavenger receptor-A on VLC each demonstrated restricted tumor growth and reduced vascular density [15,19]. However, these antibodies were against murine antigens, and therefore not directly translatable to humans.

The therapeutic effect seen with anti-CD52 therapy of ovarian tumors in mice is consistent with the aforementioned murine studies that indicate that myeloid cells promote tumor angiogenesis, vasculogenesis, and tumor growth. We observed a clear reduction in microvascular density in tumors treated with anti-CD52 therapy. This reduction in microvascular density correlated with a reduction of tumor vascular perfusion.

The observation that Alemtuzumab therapy can potently kill ovarian cancer VLC identifies a bona-fide therapeutic with which to test the importance of anti-VLC/myeloid cell therapy in human solid tumors. It is important that these studies confirmed the ability of Alemtuzumab to induce complement-mediated VLC killing within tumor ascites, an environment that closely resembles the in vivo tumor microenvironment. This would suggest that treatment effect will not be minimized by tumor-associated immunosuppressive elements or complement inhibitors. In addition, as Alemtuzumab killing is complement-mediated rather that cell-mediated, Alemtuzumab killing is less likely to be negatively impacted by dysfunctional cellular immunity. This is consistent with the activity of Alemtuzumab seen in chronic lymphocytic leukemia (CLL).

We demonstrated that Alemtuzumab can effectively kill VLC. We also observed that VLC appear to be a subset of Tie2^+ ^monocytes. Therefore, Alemtuzumab is capable of killing at least a subset of Tie2^+ ^monocytes. Furthermore, CD52 expression was identified on the vast majority of ovarian tumor-associated Tie2^+ ^monocytes, independent of their relationship to VLC, suggesting Alemtuzumab can target the majority of Tie2^+ ^monocytes. Tie2^+ ^monocytes have been reported in several solid tumor types including colorectal, breast, gastric, pancreatic, and lung carcinomas[20] Consistent with this finding, we observed VLC in melanoma, breast, lung, and ovarian cancer. Taken together, these observations suggest that Alemtuzumab may be an effective therapeutic agent targeting VLC/Tie2+ monocytes in not just ovarian cancer but various other solid tumors as well. Use of Alemtuzumab could be restricted in heavily pretreated cancer patients as the primary side effect associated with Alemtuzumab therapy is immune-suppression. However, given the unique disposition of ovarian cancer to grow in a manner restricted to the peritoneal cavity it is possible that systemic side-effects could be minimized by intraperitoneal delivery of the drug.

Despite being a well-documented therapeutic target, the exact function of CD52 remains unknown. In the ID8-VEGF tumors, VLC account for the vast majority of tumor-associated host hematopoietic cells, thus the majority of the impact is likely attributable to an anti-VLC effect. Human tumors, in contrast, are significantly more complex. CD52 expression is observed on numerous tumor infiltrating host cells including lymphocytes, neutrophils, and mast cells. Therefore it is possible Alemtuzumab could have multiple different effects via this broad targeting. In addition to the expected effects on angiogenesis based on the elimination of VLC, Alemtuzumab may also inhibit angiogenesis via the elimination of B cells and mast cells from the tumor microenvironment; both of these cell types have also been implicated in promoting angiogenesis and tumor growth [28-30].

Eliminating VLC may also impact anti-tumor immunity. Recent studies indicate that elimination of CD11c^+ ^cells, a population of cells that would include VLC, from the tumor microenvironment can actually enhance anti-tumor immunity [31]. This is consistent with an immunosuppressive phenotype of VLC [32]. Alemtuzumab could also promote anti-tumor immunity by eliminating regulatory T cells (T regs). T regs have been reported to accumulate in late stage ovarian tumors and to be a negative prognostic factor [33]. In fact, in ovarian cancer T-regs may be induced by cancer-associated myeloid cells such as VLC [34]. There is a potential detrimental immune-modulatory effect of Alemtuzumab via the elimination of anti-tumor T cells, or other inflammation mediated anti-tumor effects. However, at least in late stage tumors the impact of this anti-tumor immunity seems minimal.

## Conclusion

Given the wealth of information confirming a critical role for myeloid cells in promoting tumor growth in murine models of cancer, it is essential to determine the importance of myeloid cells in human tumor growth. Development of novel pharmaceutical agents could cost over $500,000,000 and take 10 years or longer [35]. Our data provide critical pre-clinical evidence for the use of Alemtuzumab in clinical trials as an anti-VLC, antiangiogenic therapy in ovarian cancer. The observation that VLC are present in numerous tumor types besides ovarian cancer suggests that, if not limited by immunosuppressive side effects, Alemtuzumab may be an effective therapeutic in other solid tumors. The identification of an FDA-approved agent with a significant history of clinical use will allow immediate proof-of-principle cancer therapy clinical trials in humans.

## Abbreviations

ANOVA: Analysis of Variance between Groups; CLL: Chronic Lymphocytic, Leukemia; FACS: Fluorescence activated cell sorting; FDA: Food and Drug Administration; IHC: Immunohistochemistry; MMP-9: Matrix Metalloproteinase-9; qRT-PCR: Quantitative real time polymerase chain reaction; RT-PCR: Reverse-transcription polymerase chain reaction; T regs: Regulatory T cells; VE-Cadherin: Vascular Endothelial Cadherin; VLC: Vascular leukocytes; VEGF: Vascular Endothelial Growth Factor

## Competing interests

The University of Michigan and RJB have submitted a patent regarding the use of Alemtuzumab as an anti-angiogenic agent in ovarian cancer. This was submitted after the completion of the described work.

## Authors' contributions

HP: Performed experiments, wrote manuscript, GS: Performed experiments, IS: Performed experiments, KM: Performed experiments, AK: Contributed research material, RKR: Contributed research material, GC: Contributed research material, critical reading of manuscript, JCG: Contributed research material, critical reading of manuscript, RJB: Designed and performed experiments, wrote manuscript. All authors have read and approved the final manuscript.
